# Assessing the Risk of Birth Defects Associated with Exposure to Fixed-Dose Combined Antituberculous Agents during Pregnancy in Rats

**DOI:** 10.1100/2012/585094

**Published:** 2012-05-03

**Authors:** O. Awodele, E. B. Patrick, Esther Oluwatoyin Agbaje, A. A. Oremosu, S. C. Gbotolorun

**Affiliations:** ^1^Department of Pharmacology, College of Medicine, University of Lagos, Idi-Araba 12003, Lagos, Nigeria; ^2^Department of Anatomy, College of Medicine, University of Lagos, Idi-Araba 12003, Lagos, Nigeria

## Abstract

Due to the risks of disease progression and transmission to the newborn, treatment of tuberculosis is often pursued during pregnancy and fixed-dose combined antituberculous agents have been found to be beneficial. Unfortunately, there is paucity of data on the safety of the fixed-dose combined antituberculous drugs during pregnancy. This study intends to assess the teratogenic effect of fixed-dose combined antituberculous drugs on the organogenesis stage of fetal development and also investigate the possible roles of vitamin C in modulating the teratogenic effects of these agents on the fetus using animal model. Pregnant rats were divided into 3 groups with 12 animals per group: *group 1* received distilled water (10 mL/kg) orally; *group 2* received 51.4 mg/kg/day of fixed-dose combined antituberculous agents orally; *group 3* received 51.4 mg/kg/day of fixed-dose combined antituberculous agents plus vitamin C (10 mg/kg/day) orally. Six rats in each group were randomly selected and sacrificed on day 20 by cervical dislocation prior to day 21 of gestation, and the foetuses were harvested through abdominal incision for physical examination. Blood samples were collected from the 1st filial rats of the remaining six animals for biochemical and hematological examination. The liver, kidney, heart, and brain of all the sacrificed animals were used for histopathological examination. There were significant (*P* ≤ 0.05) low birth weights of the foetuses of the animals that were treated with fixed-dose combined antituberculous agents. The haematological parameters also revealed a reduction in the platelets counts and neutrophiles at the first filial generation. Significant (*P* ≤ 0.05) elevations in the levels of aspartate aminotransferase (AST) and alkaline phosphatase (ALP) in the foetuses of the animals treated with fixed-dose combined antituberculous agents were also observed. However, the combination of vitamin C with fixed-dose combined antituberculous agents significantly (*P* ≤ 0.05) reduced the level of AST. Fixed-dose combined antituberculous agents have teratogenic potential as shown in low birth weight and mild liver damage in the first filial of the treated animals. As much as it is imminent to treat TB patients in pregnancy, there is need to always exercise caution and clinically weigh the risk-benefit ratio.

## 1. Introduction

Tuberculosis (TB) is the world's greatest infectious killer of women of reproductive age and the leading cause of death among people with HIV/AIDS. Untreated tuberculosis, however, has been associated with higher morbidity and mortality among pregnant women [1]. In 2007, an estimated 13.7 million people had active TB disease, with 9.3 million new cases and 1.8 million deaths. The annual incidence rate varied from 363 per 100,000 in Africa to 32 per 100,000 in the Americas [2].

Maternal tuberculosis has been associated with an increased risk of spontaneous abortion, perinatal mortality, and low birth weight in some studies [3]. Due to the risks of disease progression and transmission to the newborn, treatment of tuberculosis is often pursued during pregnancy [4, 5]. Almost all authors recommend some forms of aggressive treatment of active tuberculosis, given that the benefits of treatment likely outweigh the risk of fetal damage, even in the case of drugs that may have some toxicity associated with in utero exposure [6]. One of the major problems militating against the management of tuberculosis is the lack of compliance to medication by the infected patients as a result of multidrug needed to be taken daily leading to resistance [7]. Thus, the World Health Organization (WHO) and the International Union against Tuberculosis and Lung Disease (IUATLD) recommend the replacement of single-drug preparations by fixed-dose combination therapy (FDCs) [8]. The justification for this recommendation is that FDCs provide a simple approach to delivering the correct number of drugs at the correct dosage as all the necessary drugs are combined in a single tablet [9]. Combinations of two to four anti-TB drugs administered in fixed doses in a single tablet decrease the risk of developing multidrug-resistant tuberculosis [10], and fixed-dose drug combinations have been found to be beneficial and cost-effective treatment for TB [11].

Quoting data from the old literature, the use of ethionamide and prothionamide during pregnancy remains controversial. In rodents, high doses of ethionamide have been associated with growth retardation, abortions, and malformations, including CNS defects [12]. In human studies, 2 reports reviewing 47 cases failed to observe deleterious effects of ethionamide [13]. On the other hand, Potworowska et al. [14] encountered congenital malformations in 7 of 23 children exposed to ethionamide. The work of Schardein [15], with approximately 70 case reports, found no teratogenic effects with these drugs. Many authors still recommend withholding their use in pregnancy [16], although some recognize the need to use these drugs if the risk of active disease outweighs potential risks to the fetus [3].

A number of theoretical mechanisms have suggested potential modification of fetal toxicity (as seen in combination therapies) to include the induction of biochemical or metabolic changes (with negative consequences on the fetus) in the maternal system that would not have arisen in the use of a single drug or the combination increasing the dose available to interact with fetal mechanism leading to negative effects or changes that are absent in the use of the drug singly. An important mechanism of induction of fetal toxicity by teratogenic agents is the induction of oxidative stress in the fetal system [17]. Oxidative stress can cause cell death, and even moderate oxidation can trigger apoptosis, while more intense stresses may cause necrosis [18].

Inferring from the above facts, it suffices to say there is little and inconsistent data on the safety of the fixed-dose combined antituberculous drugs during pregnancy. Therefore, this study intends to assess the teratogenic effect of fixed-dose combined antituberculous drugs on the organogenesis stage of fetal development and also investigate the possible roles of vitamin C (an antioxidant) in modulating the teratogenic effects of these agents on the fetus using animal model. The findings obtained from this study may give a directional surveillance in humans and possible generation of more reliable and consistent data.

## 2. Methodology

### 2.1. Drugs

Fixed-dose combined antituberculous (TB) agents contain rifampicin 150 mg, isoniazid 75 mg, pyrazinamide 400 mg and Ethambutol 275 mg per caplet. The fixed-dose combination drugs were obtained from Direct Observed Treatment (DOT) Clinic in Lagos University Teaching Hospital, Lagos-Nigeria. Ascorbic acid (Vitamin C Tablets (100 mg per tablet)) was obtained from the outpatient Pharmacy Department of the Lagos University Teaching Hospital, Lagos-Nigeria.

### 2.2. Animals

Sexually mature adult Albino rats (male and female) with average weight of 160 g were obtained from the Laboratory Animal Centre of College of Medicine, University of Lagos, Nigeria. The animals were authenticated in Zoology Department, Faculty of Science, University of Lagos, Nigeria. They were made to acclimatize for two weeks before the commencement of the experiment. The animals were fed on Pfizer Animal Feed cubes and water ad libitum. The investigation conforms to the Guide for the Care and Use of Laboratory Animals published by the U. S. National Institutes of Health (NIH Publication No. 85-23, revised 1996) for studies involving experimental animals. Ethical permission for use of animals for this research was obtained from the College of Medicine, University of Lagos, Research Ethics Committee.

### 2.3. Cycle Determination

After two weeks of acclimatization, vaginal smears of female rats were done for viewing under the microscope to determine the females that will be receptive to the males during mating. Vaginal secretion was collected (in the morning between 8:00 and 9:00 a.m.) with a plastic pipette filled with 10 *μ*L of normal saline by inserting the tip into the rat vagina, but not deeply. Vaginal fluid was then placed on glass slides to observe under a light microscope (×10 magnification) according to the method of Marcondes et al. [19]. The proestrus stage is the receptive state that was microscopically checked out for [20], and the virginal smears of mated female rats were assessed for the presence of sperm plug. The first day to see sperm plug was taken as day 1 of pregnancy.

### 2.4. Treatment Groups

Rats have average gestational period of 21 days. Pregnant rats were divided into 3 groups with 12 animals per group. The therapeutic doses of drugs were administered to simulate the treatment pattern in DOT Clinic of the University of Lagos Teaching Hospital (LUTH), Lagos, Nigeria

*Group 1* received distilled water (10 mL/kg) orally.
*Group 2* received 51.4 mg/kg/day of fixed dose combined antituberculous agents orally.
*Group 3* received 51.4 mg/kg/day of fixed dose combined antituberculous agents plus vitamin C (10 mg/kg/day) orally.

### 2.5. Morphological and Histopathological Examination

Six rats in each group were randomly selected, subjected to light ether anesthesia, and sacrificed on day 20 by cervical dislocation prior to day 21 of gestation, and the foetuses were harvested through abdominal incision for physical examination. The tail length, crown-rump length, umbilical cord length, total weight (includes weight of fetus and placenta), and weights of fetus were recorded. After the physical examination, the harvested foetuses were taken for gross histopathological analysis at the Morbid Anatomy Department of the College of Medicine, University of Lagos. The histopathological features observed were formation of digital rays, neural tube defects, cleft palate, and general growth abnormalities.

### 2.6. Biochemical and Haematological Examination

The six other rats in each group were allowed to litter, and the litters were allowed to grow for a period of at least one month after which six animals from each litter were randomly selected. Blood samples were collected from the animals for biochemical and hematological examination. The fully automated clinical chemistry analyzer (Hitachi 912, Boehringer Mannheim, Germany) was used to determine the levels of aspartate aminotransferase (AST), alanine aminotransferase (ALT), alkaline phosphatase (ALP), urea, creatinine, total protein, cholesterol, and triglyceride; fully automated clinical haematological analyzer (Pentra-XL 80, Horiba ABX, USA) was also used to determine the levels of white blood cells, red blood cells, hemoglobin, hematocrit (packed cell volume), platelet, lymphocyte count, and percentage neutrophiles.

### 2.7. Histopathological Examination

The liver, kidney, heart, and brain of all the sacrificed animals were fixed in 10% formalin in labeled bottles. Tissues were processed routinely and embedded in paraffin wax. Sections of 5 *μ* thickness were cut, stained with haematoxylin and eosin, and examined under the light microscope by a pathologist.

### 2.8. Statistical Analysis

Results were expressed as mean ± SEM. The data were subjected to one-way analysis of variance (ANOVA) test, and differences between samples were determined by Dunnett's multiple comparison test, using the Graph Pad Prism (statistical) software. Results were considered to be significant at *P* ≤ 0.05.

## 3. Results

Table 1 results showed 100% live foetuses and 0% resorption with average litter number of 7.33 ± 0.023 (group 1), 8.33 ± 0.43 (group 2), and 8.00 ± 0.00 (group 3).

There were significant (*P* ≤ 0.05) reductions in the body weight and placenta weight of animals that received therapeutic doses of combined fixed-dose antituberculous agents (group 2) and combined fixed-dose antituberculous agents plus vitamin C (group 3) compared with the control group (Table 2). The crown rump length, umbilical cord length, and tail length showed no significant (*P* ≥ 0.05), difference across groups. The gross pathology (morphology) results of the foetuses of treated animals revealed no abnormalities (Table 3).

There were significant (*P* ≤ 0.05) reductions in the platelet counts between the pups of animals that received therapeutic doses of combined fixed-dose antituberculous agents (group 2) and combined fixed-dose antituberculous agents plus vitamin C (group 3) compared with the control group. The neutrophiles results of group 2 (0.8100 ± 0.1643) significantly reduced compared with the control group and combined fixed-dose antituberculous agents plus vitamin C (group 3). There were no significant differences (*P* ≥ 0.05) in the white blood cells, red blood cells, haematocrit, and haemoglobin results across the groups (Table 4).

Table 5 shows a significant increase (*P* ≤ 0.05) in the values of AST (213.3 ± 16.35) of the pups of animals that received therapeutic doses of combined fixed-dose antituberculous agents (group 2) compared with control group (151.3 ± 12.25). There was a subsequent reduction (*P* ≤ 0.05) in the AST value of animals that received combined fixed-doses antituberculous agents plus vitamin C compared with group 2. A significant increase was observed in the urea levels of the pups of animals that received therapeutic doses of combined fixed-dose antituberculous agents (group 2) and combined fixed-dose antituberculous agents plus vitamin C (group 3) compared with the control group. There was also a significant decrease in the level of total protein of the pups of animals that received therapeutic doses of combined fixed-dose antituberculous agents and combined fixed-dose antituberculous agents plus vitamin C compared with the control group. The pups of the animals that received therapeutic doses of combined fixed-dose antituberculous agents showed an elevated level of ALP compared with the control group (171.9 ± 3.65). However, there was a remarkable increase (*P* ≤ 0.05) in the ALP level of the pups of animals that received combined fixed-dose antituberculous agents plus vitamin C (287 ± 55.3) compared with the pups of animals that received combined fixed-dose antituberculous agents (211.6 ± 12.9).

**Figure 1:**
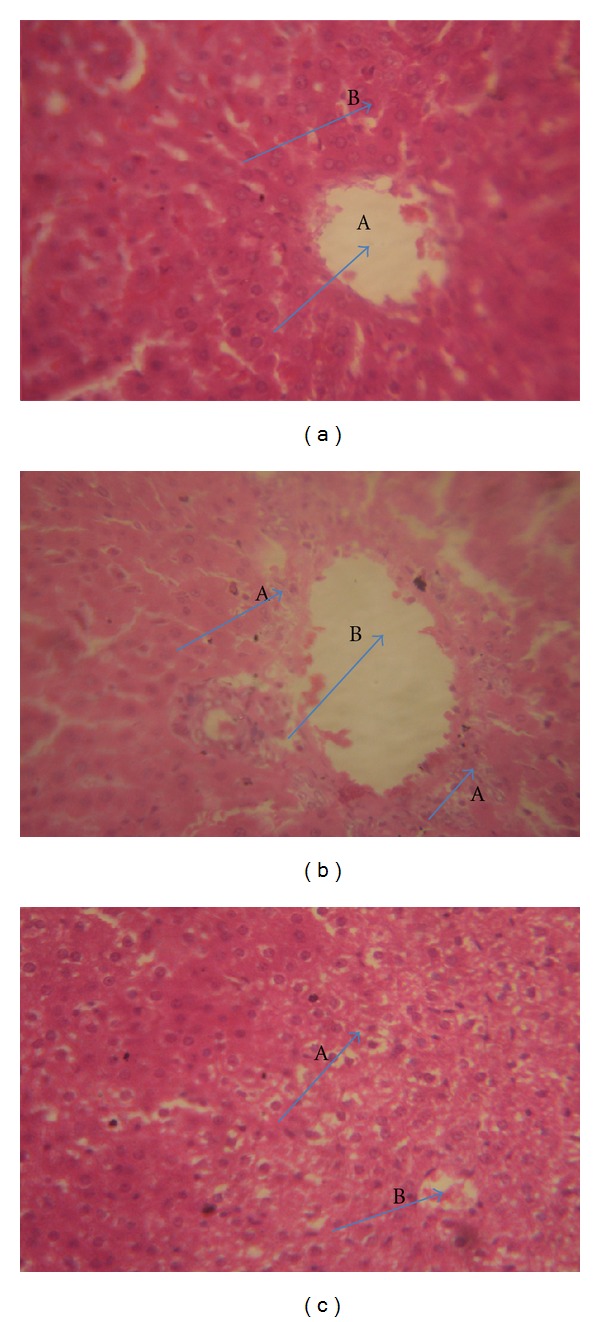
(a) The micrograph of the liver section obtained from first filial control rats, magnification ×400. (A) Hepatic central vein, (B) normal hepatocyte. (b) The micrograph of the liver section obtained from first filial rats treated with fixed-dose combined antituberculous agents, magnification ×400. (A) Hepatic necrosis, (B) hepatic central vein. (c) The micrograph of the liver section obtained from first filial rats treated with fixed-dose combined antituberculous agents plus vitamin C, mag ×40. (A) Hepatocytes with intracytoplasmic inclusions, (B) hepatic central veins.

**Figure 2:**
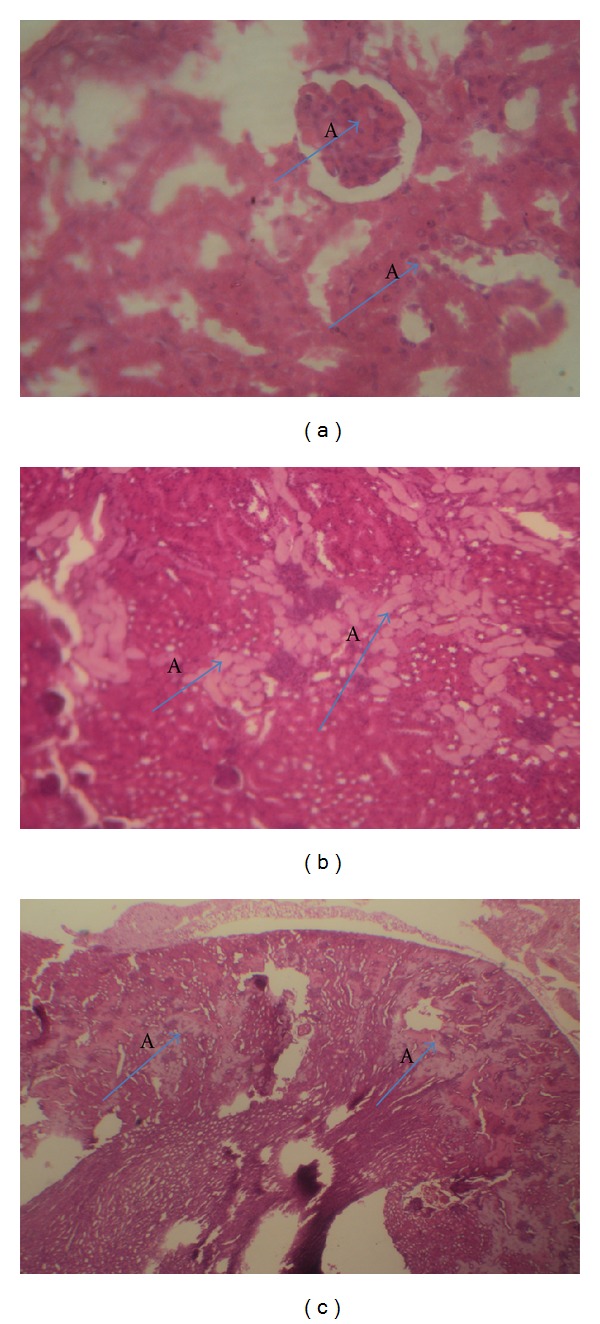
(a) The micrograph of the kidney section obtained from first filial control rats, magnification ×400. (A) Normal glomerulus. (b) The micrograph of the kidney section obtained from first filial rats treated with fixed-dose combined antituberculous agents, mag ×40. (A) Acute tubular necrosis. (c) The micrograph of the kidney section obtained from first filial rats treated with fixed-dose combined antituberculous agents plus vit C, mag ×40. (A) Acute tubular necrosis.

Figure 1 showed the histology slides of the liver cells. There were focal areas of hepatocyte necrosis seen in the group treated with fixed-dose combined antituberculous agents (1b) and hepatocytes with intracytoplasmic inclusions in the group treated with fixed-dose combined antituberculous agents plus vitamin C (1c). There was acute tubular necrosis seen in the kidney cells of both the group treated with the fixed-dose combined antituberculous agents (2b) and that treated with fixed dose combined antituberculous agents plus vitamin C (2c) (Figure 2).

## 4. Discussion

It is a general principle that the administration of any drug to a pregnant patient is to be avoided, because of possible fetal damage. However, the treatment of TB in pregnancy has been advocated; given that the benefit of treatment likely outweighs the risk of fetal damage, even in the case of drugs that may have some toxicity associated with in utero exposure [6]. In the last decade, the conventional single-drug preparations used in the management of TB have been replaced by fixed-dose combination (FDC) therapy [8]. The studies of Tasduq et al. [21] and Awodele et al. [22] have documented the hepatotoxicity of isoniazid and rifampicin. However, there is little and controversial data on the teratogenicity of fixed-dose combined antituberculous agents.

The present study showed that fixed-dose combined antituberculous agents had no effect on the morphology (physical appearance) of the foetuses. However, there was a significantly low birth weight of the foetuses of the animals that were treated with fixed-dose combined antituberculous agents. This observation may infer that children born from mothers that have undergone tuberculosis treatment during pregnancy using fixed-dose combined antituberculous agents have the tendency of having stunted growth, slow cognitive development, and chronic disease later in life [23]. Studies have also proposed that children with low birth weight have an increased risk of developing diabetes [24, 25], obesity [26], and reduced intelligence [27] later in life.

The haematological parameters revealed a reduction in the platelets counts and neutrophiles of the animals treated with antituberculous agents at the first filial generation. The low neutrophiles and platelets may indicate that the use of fixed-dose antituberculous agents in pregnancy could result in immune suppression and bleeding disorder of the fetuses, respectively. There were also significant (*P* ≤ 0.05) elevations in the levels of aspartate aminotransferase (AST) and alkaline phosphatase (ALP) in the fetuses of the animals treated with fixed-dose combined antituberculous agents. These elevations may indicate liver damage in the first filial generation [28, 29]. The pups of animals that were treated with combination of vitamin C and fixed-dose antituberculous agents showed a significant reduction in the AST level compared with the pups of animals that received only antituberculous agents. This result may underscore the modulatory ability of vitamin C against the fixed-dose combined antituberculous agents effect on inducing hepatic damage, and it corroborates the earlier finding of Awodele et al. [22]. The histopathological examination of the hepatic cells corroborated the liver enzymes results. There were focal areas of hepatocyte necrosis seen in the fixed-dose-combined-antituberculous-treated group, however, only hepatocytes with intracytoplasmic inclusions were seen in the group treated with fixed-dose antituberculous agents plus vitamin C. The histology of the kidney cells also revealed acute tubular necrosis in both the group treated with fixed-dose combined antituberculous agents and that treated with fixed-dose combined antituberculous agents plus vitamin C. Although the blood chemistry results did not show a significant difference (*P* ≤ 0.05) in the level of creatinine of the group treated with fixed-dose combined antituberculous agents compared with control, a significant elevation in the level of urea of the group treated with fixed-dose combined antituberculous agents was observed; the clinical correlation of these results may indicate renal injury.

## 5. Conclusion

Fixed dose combined antituberculous agents have teratogenic potential as shown in low birth weight, mild liver and kidney damage in the first filial of the treated animals. As much as it is imminent to treat TB patients in pregnancy, there is need to always exercise caution and clinically weigh the risk-benefits ratio. Options of induced abortion may be considered in patients with TB during pregnancy in order to avoid unwanted congenital malformations.

## Figures and Tables

**Table 1:** Incidence of resorption and live foetus in treated rats.

Groups	Stage of drug exposure	Percentage (%) of live foetus	Average number of live foetus	Number of resorption
Group 1	Organogenesis	100	7.33 ± 0.023	0
Group 2	Organogenesis	100	8.33 ± 0.43	0
Group 3	Organogenesis	100	8.00 ± 0.00	0

Group 1: control group. Group 2: administered with clinical dose (51.4 mg/kg/day) of fixed-dose combined antituberculous agents. Group 3: administered with clinical dose of fixed-dose combined antituberculous agents plus 10 mg/kg/day of Vitamin C.

**Table 2:** Incidence of growth retardation and size abnormality in fetuses of treated rats.

Groups	SE	BW + PW (g) (Mean ± SEM)	BW (g) (Mean ± SEM)	PW (g) (Mean ± SEM)	CRL (cm) (Mean ± SEM)	UCL (cm) (Mean ± SEM)	TL (CM) (Mean ± SEM)
Group 1	OR	4.793 ± 0.6750	4.173 ± 0.6165	0.6205 ± 0.01799	4.583 ± 0.1222	2.967 ± 0.1453	1.267 ± 0.04216
Group 2	OR	4.181 ± 0.1340*	3.567 ± 0.06215*	0.4818 ± 0.01337*	4.733 ± 0.09189	2.883 ± 0.1621	1.200 ± 0.05164
Group 3	OR	3.638 ± 0.233*	3.075 ± 0.1341*	0.6233 ± 0.03565*	4.183 ± 0.2469	2.800 ± 0.1317	1.117 ± 0.05426

SE: stage of exposure, OR: organogenesis, BW + PW: body weight + placenta weight, BW: body weight, PW: placenta weight, CRL: crown-rump length, UCL: umbilical cord length, TL: tail length.

*P ≤ 0.05 compared with group 1 (control).

Group 1: control group. Group 2: administered with clinical dose (51.4 mg/kg/day) of fixed-dose combined antituberculous agents. Group 3: administered with clinical dose of fixed-dose combined antituberculous agents plus 10 mg/kg/day of Vitamin C.

**Table 3:** Gross pathological (morphology) results of fetuses of treated animals.

Groups	Stage of exposure	No. of foetus use	Formation of digital rays	Neural tube defect	Cleft palate	General growth abnormalities	Abnormal heart	Abnormal liver	Abnormal brain	Abnormal kidney
Group 1	ORG	18	0/18	0/18	0/18	0/18	0/18	0/18	0/18	0/18
Group 2	ORG	18	0/18	0/18	0/18	0/18	0/18	0/18	0/18	0/18
Group 3	ORG	18	0/18	0/18	0/18	2/18	0/18	0/18	0/18	0/18

ORG: organogenesis.

Group 1: control group. Group 2: administered with clinical dose (51.4 mg/kg/day) of fixed-dose combined antituberculous agents. Group 3: administered with clinical dose of fixed-dose combined antituberculous agents plus 10 mg/kg/day of Vitamin C.

**Table 4:** Hematological Profile of Rats at first filial.

Groups	SOE	NOF	WBC (Mean ± SEM)	RBC (Mean ± SEM)	HGB (Mean ± SEM)	HCT (Mean ± SEM)	PCT (Mean ± SEM)	LYPM (Mean ± SEM)	NEU (Mean ± SEM)
Group 1	ORG	6	5.433 ± 0.2319	6.677 ± 0.1444	13.70 ± 0.2646	40.92 ± 0.6519	792.5 ± 18.92	3.705 ± 0.2207	1.345 ± 0.1276
Group 2	ORG	6	4.983 ± 1.101	6.330 ± 0.2752	11.97 ± 0.7864	38.83 ± 1.456	372.7 ± 72.42*	3.722 ± 0.8835	0.8100 ± 0.1643*
Group 3	ORG	6	6.517 ± 0.7510	6.108 ± 0.3862	11.98 ± 0.695	37.17 ± 1.615	475.7 ± 100.7*	4.717 ± 0.4642	1.622 ± 0.2774

SOE: stage of exposure, NOF: number of animals, ORG: organogenesis, WBC: white blood cell count, RBC: red blood cell count, HGB: hemoglobin count, HCT: haematocrit, PCT: platelet count, LYPM: lymphocyte, NEU: neutrophiles.

*P ≤ 0.05 compared with group 1 (control).

Group 1: control group. Group 2: administered with clinical dose (51.4 mg/kg/day) of fixed-dose combined antituberculous agents. Group 3: administered with clinical dose of fixed-dose combined antituberculous agents plus 10 mg/kg/day of Vitamin C.

**Table 5:** Blood chemistry Results at first filial.

Groups	SOE	*N*	AST (Mean ± SEM)	CREA (Mean ± SEM)	ALT (Mean ± SEM)	UREA (Mean ± SEM)	T. PRO (Mean ± SEM)	CHOLES (Mean ± SEM)	TG (Mean ± SEM)	ALP (Mean ± SEM)
Group 1	ORG	6	151.3 ± 12.25	38.02 ± 1.285	71.30 ± 7.433	4.873 ± 0.280	72.17 ± 1.497	2.217 ± 0.079	0.8167 ± 0.048	171.9 ± 3.65
Group 2	ORG	6	213.3 ± 16.35*	33.12 ± 1.285	57.78 ± 4.89	8.73 ± 1.746*	65.88 ± 2.18*	2.03 ± 0.27	0.50 ± 0.037*	211.6 ± 12.9*
Group 3	ORG	6	163.6 ± 16.09^*α*^	23.86 ± 2.31	53.03 ± 12.39	8.950 ± 0.295*	49.30 ± 1.39^*α*^	2.052 ± 0.11	0.86 ± 0.051	287 ± 55.3^*α*^

SOE: stage of exposure, ORG: organogenesis, N: number of animals, AST: aspartate aminotransferase, CREA: creatinine, ALT: alanine aminotransferase, T.PRO: total protein, CHOLES: cholesterol, TG: triglyceride, ALP: alkaline phosphatase.

*P ≤ 0.05 compared with group 1 (control).

^*α*^ *P* ≤ 0.05 compared with group 2.

Group 1: control group. Group 2: administered with clinical dose (51.4 mg/kg/day) of fixed-dose combined antituberculous agents. Group 3: administered with clinical dose of fixed-dose combined antituberculous agents plus 10 mg/kg/day of Vitamin C.
